# Vaginal Metastasis of Colorectal Cancer Treated With Image-Guided Intracavitary-Interstitial Brachytherapy: A Case Report

**DOI:** 10.7759/cureus.79253

**Published:** 2025-02-18

**Authors:** Soichiro Sakamoto, Ken Ando, Takuya Kumazawa, Takahiro Oike, Tatsuya Ohno

**Affiliations:** 1 Department of Radiation Oncology, Gunma University Graduate School of Medicine, Maebashi, JPN

**Keywords:** colorectal cancer, image-guided brachytherapy, intracavitary-interstitial brachytherapy, oligometastasis, radiation therapy, vaginal metastasis

## Abstract

Metastasis of a colorectal primary tumor to the vagina is an uncommon condition, and its treatment strategies have not been well established. Computed tomography (CT)-guided intracavitary-interstitial (IC-IS) brachytherapy uses a few interstitial needles, in addition to conventional intracavitary applicators (such as the Fletcher-Suit applicator or vaginal cylinder), and achieves conformal dose distribution to bulky and/or irregularly shaped tumors. Here, we report a first case of an oligometastatic vaginal tumor from the colorectum that was treated successfully with radiotherapy using CT-based IC-IS brachytherapy. External beam radiotherapy (EBRT), followed by four sessions of IC-IS brachytherapy, resulted in a satisfactory dose prescription to the target while sparing normal organs adjacent to the tumor. This led to radiological complete remission of the tumor over at least six months, with no severe adverse effects. This case indicates that radiotherapy using CT-based IC-IS brachytherapy may be a viable treatment option for metastatic vaginal tumors.

## Introduction

Metastasis of a colorectal primary tumor to the vagina is a rare malignancy with no standard treatment [1]. If the disease is oligometastatic, local therapy such as surgical resection [2-12] or radiotherapy [1,13-16] can be considered. High-dose-rate brachytherapy is one of the treatment options since it is used as the standard treatment for primary vaginal tumors [17]. In 2011, we reported a computed tomography (CT)-guided intracavitary-interstitial (IC-IS) brachytherapy procedure in which a few interstitial needles were added to conventional intracavitary applicators (i.e., the Fletcher-Suit applicator or vaginal cylinder) to achieve conformal dose distribution within bulky and/or irregularly shaped tumors [18]; however, there is no report describing the use of IC-IS brachytherapy to treat metastatic vaginal tumors. Here, we report a case of a vaginal tumor that metastasized from the colorectum, which was treated successfully with radiotherapy using CT-based IC-IS brachytherapy.

## Case presentation

**Figure 1:**
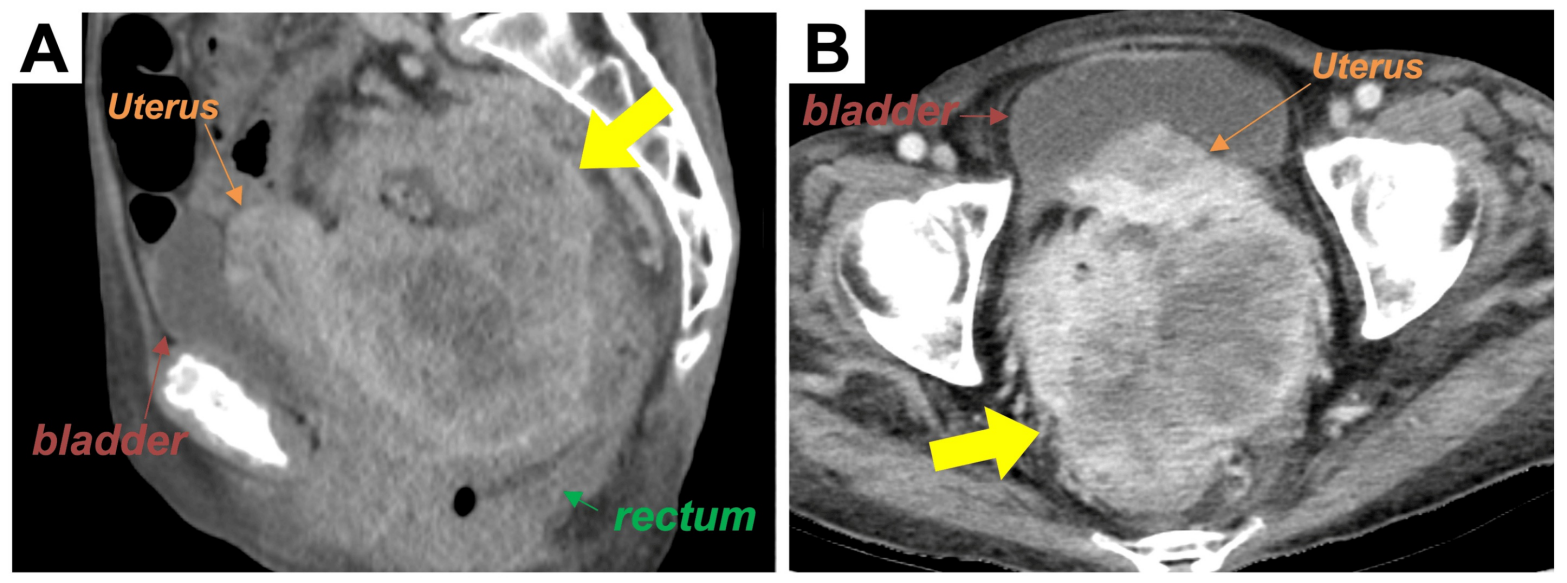
Baseline computed tomography images of the primary tumor (A) Sagittal plane. (B) Axial plane. Yellow arrows indicate the tumor.

A 78-year-old woman with sigmoid colon cancer (adenocarcinoma, cT4bN0M0 cStage IIC) with uterine invasion (Figure 1) underwent three courses of neoadjuvant chemotherapy (levofolinate, oxaliplatin, 5-fluorouracil, and panitumumab) aiming for tumor volume reduction.

The tumor showed a 16% reduction in diameter, leading to the surgeons' consideration that the tumor is resectable. Surgery comprised low anterior resection of the rectum with D3 lymphadenectomy and hysterectomy with bilateral salpingo-oophorectomy. The tumor was diagnosed as adenocarcinoma (negative for *RAS*/*BRAF* mutations, *HER2* amplification, and microsatellite instability), sT4bN0M0 sStage IIC. The pathological resection margin was negative. The patient continued the chemotherapy up to 17 courses; however, during the chemotherapy, an asymptomatic mass on the posterior vaginal wall emerged, which was diagnosed pathologically as a metastasis of the colorectal cancer (adenocarcinoma positive for CDX2 and negative for PAX8 by immunohistochemistry). No other metastasis was observed radiologically. Due to advanced age and a history of surgery on the primary tumor, the patient selected radiotherapy for the definitive treatment of the vaginal tumor. The patient was referred to our Department of Radiation Oncology 20 months post-surgery.

**Figure 2:**
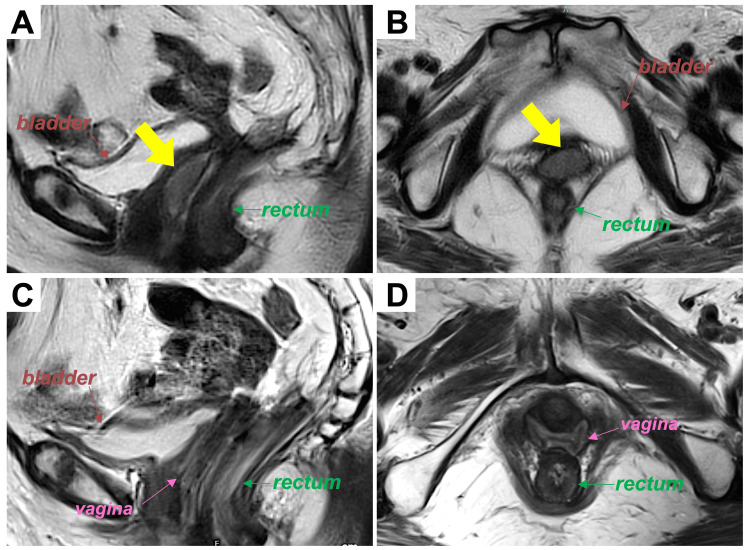
T2-weighted magnetic resonance images of the metastatic vaginal tumor before or six months after radiotherapy (A) Sagittal plane before radiotherapy. (B) Axial plane before radiotherapy. (C) Sagittal plane six months after radiotherapy. (D) Axial plane six months after radiotherapy. Yellow arrows indicate the tumor.

The vaginal tumor was located 1 cm from the vaginal introitus and measured 35 × 25 × 11 mm (Figure 2A, 2B).

**Figure 3:**
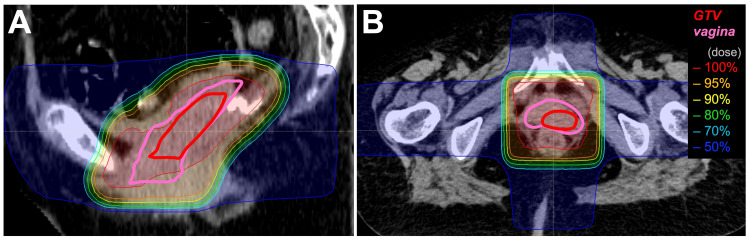
Dose distribution for external beam radiotherapy (A) Sagittal plane. (B) Axial plane. GTV: gross tumor volume

The tumor was treated with external beam radiotherapy (EBRT) followed by brachytherapy. Concurrent chemotherapy was avoided considering the patient's advanced age. EBRT comprised a total dose of 30 Gy, delivered in 15 fractions (five fractions per week), to the vagina using a box four-field irradiation technique (Figure 3).

The vagina and the tumor were identified by CT, in which magnetic resonance imaging (MRI) was registered. The gross tumor volume (GTV) encompassed the tumor. The clinical target volume (CTV) encompassed the entire vagina. The planning target volume (PTV) margin was 0.5, 1.0, and 1.5 cm in a right-left, superior-inferior, and anterior-posterior direction. The internal target volume was not defined. Elective nodal irradiation was not performed due to the fact that the vaginal tumor was free from paravaginal tissue involvement, as assessed by internal examination, and it was oligometastatic after the continuation of chemotherapy for over 20 months.

**Figure 4:**
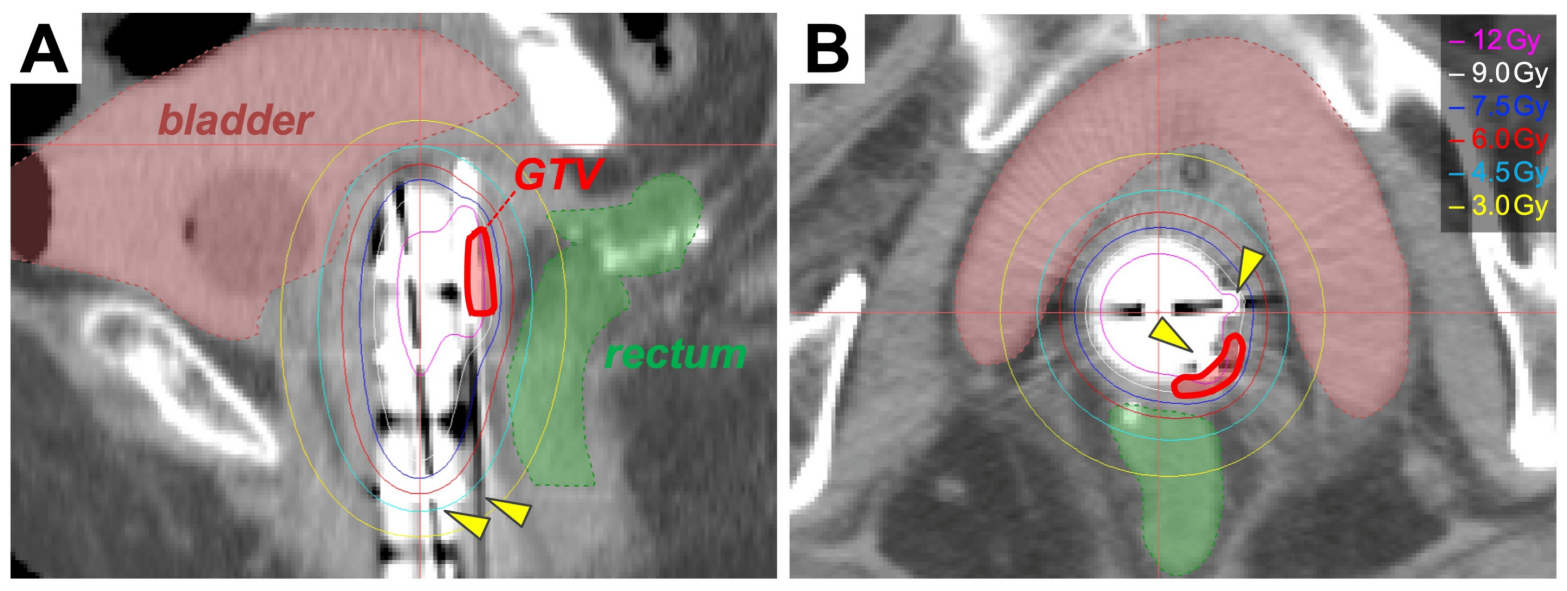
Dose distribution for intracavitary-interstitial brachytherapy (A) Sagittal plane. (B) Axial plane. Yellow arrowheads indicate needles. GTV: gross tumor volume

After the completion of EBRT, the tumor showed a 23% reduction in diameter in MRI. No toxicity was observed. Brachytherapy was performed using a high-dose-rate ^192^Ir remote-after-loading system (microSelectron, Elekta, Stockholm, Sweden) and in-room computed tomography-based three-dimensional treatment planning systems (Oncentra, Elekta, Stockholm, Sweden). A vaginal cylinder and two interstitial needles were used to target the vaginal tumor (Figure 4).

The needles were used based on the recommendation from the American Brachytherapy Society consensus guidelines for interstitial brachytherapy for vaginal cancer [17] in which the interstitial approach is recommended for tumors with a thickness greater than ~5 mm. We chose to employ the IC-IS approach with the assumption that the addition of needles to a vaginal cylinder enables conformal dose delivery to a tumor with a thickness greater than 5 mm located unevenly on the left posterior wall of the vagina. Treatment planning was performed based on in-room CT images, with guidance from MRI images obtained prior to brachytherapy. In addition, an internal examination was performed intensely to identify the location of the tumor. The GTV was defined as the tumor. Since there are no established guidelines in the literature mandating the delineation of the CTV for brachytherapy to treat metastatic vaginal tumors, we regarded the GTV as being equal to high-risk CTV. Four sessions were performed in three weeks. The dose constraint for the bladder and rectum was set at 90 Gy and 75 Gy, respectively, as the total equivalent dose in 2 Gy fractions (EQD2) with an α/β ratio of 3. Consequently, the D_90_ value (i.e., the minimum dose at which 90% of the tumor volume is irradiated) for the GTV exceeded 6 Gy in each of the four sessions, resulting in the delivery of a total EQD2 (with an α/β ratio of 10) for EBRT plus brachytherapy of 75.8 Gy (Table 1).

**Table 1:** Dose volume parameters for brachytherapy BT: brachytherapy, D_90_: minimum dose at which any 90% of the volume is irradiated, D_2 cm3_: minimal dose to the most exposed 2 cm^3^ of the volume, EBRT: external beam radiotherapy, EQD_2_: total equivalent dose in 2 Gy fractions with an α/β ratio of 10 (tumor) or 3 (normal tissues), GTV: gross tumor volume

Parameters	BT session				EQD_2_	
	1	2	3	4	BT	EBRT+BT
GTV D_90_ (Gy)	7.41	7.90	9.03	6.55	45.89	75.89
Bladder D_2 cm3_ (Gy)	4.36	5.25	4.82	5.47	31.89	61.89
Rectum D_2 cm3_ (Gy)	5.86	5.68	4.95	5.21	36.67	66.67

For the bladder and rectum, the D_2 cm3_ value (i.e., the minimal dose to the most exposed 2 cm^3^ of the volume) was below 7 Gy and 6 Gy, respectively, during each of the four sessions. The overall treatment time was 35 days. Dermatitis and vaginal inflammation (both assessed as Grade 1 according to the Common Terminology Criteria for Adverse Events version 4) were noted as acute adverse effects. At six months post-radiotherapy, the patient shows no evidence of local recurrence or late adverse effects (Figure 2C, 2D).

## Discussion

Vaginal metastasis from a primary colorectal tumor is extremely rare [8,13]; therefore, the treatment has not been standardized. To the best of our knowledge, this is the first study to describe the treatment of a metastatic vaginal tumor from a colorectal primary using CT-based IC-IS brachytherapy. In previous case reports, surgery was the most common choice of treatment with curative intent [2-12]; in some cases, neoadjuvant chemotherapy [3] or adjuvant radiotherapy (e.g., 60 Gy to the pelvic cavity) [14] was used in combination with surgery. In our case, radiotherapy (a less invasive treatment option than surgery) was selected due to the patient's advanced age and history of surgery on the primary tumor. A few papers report radiotherapy for a metastatic vaginal tumor derived from a colorectal primary. As the first, in 1966, Raider reported four cases treated with cobalt-60 or radium [16]. In 2006, Marchal et al. reported a case of metastatic vaginal tumor (5 × 4 cm) treated with radiotherapy [15]. In that report, the patient received EBRT (30 Gy) targeting the perineal regions, and the tumor showed a partial response within two months. This was followed by the after-loading of 192Ir implants (35 Gy). Nevertheless, the tumor persisted for four months; therefore, it was salvaged by surgical resection. In 2021, Ansari et al. reported a case of metastatic vaginal tumor (1.5 cm in size) treated with concurrent chemoradiotherapy [1]. In that report, the patient received intensity-modulated radiation therapy (a total of 45 Gy delivered in 25 fractions) targeting the vagina and pelvic/inguinal lymph node regions, followed by high-dose brachytherapy using a vaginal cylinder (a total of 12 Gy delivered in two fractions). The total EQD_2_ to the tumor was 83.9 Gy, and the tumor was controlled over a follow-up period of two years. In our case, we limited the irradiation field for EBRT (30 Gy) to the vagina, without including the prophylactic lymph node regions, due to the fact that the vaginal tumor was oligometastatic after continuation of chemotherapy for over 20 months. Taken together, these reports suggest that the optimal dosing and targeting regimens for this disease warrant further investigation.

During image-guided brachytherapy, we followed the dose constraints for uterine cervical cancer [19]; this led to a total EQD_2_ of 75.8 Gy while keeping the dose constraints for the bladder and rectum. We chose to employ the IC-IS approach because of the following reasons: (i) it is recommended that an interstitial approach should be considered to treat the vaginal tumors with brachytherapy when the tumor thickness is greater than ~5 mm [17] and (ii) we considered that the addition of needles to a vaginal cylinder enables conformal dose delivery to a tumor located unevenly on the left posterior wall of the vagina. As a result, we were able to deliver a total EQD_2_ greater than 70 Gy, which is required to control vaginal tumors [17,20], to a tumor measuring 35 × 25 × 11 mm. The tumor was controlled for at least six months, and the adverse effects during the acute phase were negligible. These data indicate that radiotherapy using CT-based IC-IS brachytherapy may be a viable treatment option for metastatic vaginal tumors, although longer follow-up is necessary. Besides, the IC-IS brachytherapy described in this paper may also be effective for oligometastatic disease that develops after standard-of-care treatment.

## Conclusions

Metastasis of a colorectal primary tumor to the vagina is a rare malignancy with no standardized treatment. CT-guided IC-IS brachytherapy achieves conformal dose distribution to bulky and/or irregularly shaped tumors. This is the first case of a metastatic vaginal tumor originating from a colorectal primary cancer, which was treated with radiotherapy that combined EBRT and CT-based IC-IS brachytherapy. The treatment achieved a total EQD_2_ exceeding 75 Gy, which is considered sufficient for tumor control. The patient experienced only mild adverse effects, and local tumor control was observed for at least six months post-treatment. These findings suggest that CT-based IC-IS brachytherapy could be a viable and less invasive therapeutic option for metastatic vaginal tumors, warranting further investigation with extended follow-up.
